# Supplementary material for: Scalable inference of transcriptional kinetic parameters from MS2 time series data
Source: Bioinformatics. 2021 Nov 12;38(4):1030–6. doi: 10.1093/bioinformatics/btab765 (PMC8796374; doi:10.1093/bioinformatics/btab765)
Supplement: btab765_supplementary_data [file btab765_supplementary_data.pdf]

Supplementary material for 'Scalable inference of transcriptional kinetic parameters from MS2 time series data'

Jonathan R. Bowles[1], Caroline Hoppe[1], Hilary L. Ashe[1] and Magnus Rattray[1]

[1]Faculty of Biology, Medicine and Health, University of Manchester, Manchester, M13 9PT, UK

1 Estimating single-cell parameters

The *burstinfer* toolbox provides the ability to infer single-cell transcriptional parameters, as opposed to only a single set of parameters for an entire dataset or large subsets of cells. A model is initially trained using the whole dataset or subsets of the data (e.g. spatial domains). The learned parameters are then used to infer the most likely sequence of promoter states for each cell, i.e. the sequence of 1's and 0's that generated the observed data. Inferring single-cell parameters involves high levels of uncertainty, since very little data is available to estimate the transition frequencies for one cell. However, the aim is to help visualise the spatial trend at the single-cell level that may not be apparent from looking at a single spatial region or a small number of spatial regions side-by-side. This can be achieved by spatial smoothing of the single-cell estimates to infer the mean parameter change trend.

To give an example of how the single-cell parameters can be used to visualise spatial trends, figure S1 shows the result of smoothing single-cell parameters for the *Drosophila* gene *ush*. Three separate models were trained for the outer, intermediate and central spatial regions of the embryo. These were then used to generate single-cell parameters. The left and right panels show $p_{\mathrm{off}\to\mathrm{on}}$ and $p_{\mathrm{on}\to\mathrm{off}}$, respectively: the probability of off-to-on and on-to-off transitions across the entire trace for each cell. The *loess* function from the Python *scikit-misc* library was used to smooth the data, as shown by the red curves. While there are large variations associated with the single-cell estimates, it is possible to visualise general spatial trends in the data, such as the decrease in the probability of promoter activation in cells further from the embryo midline as well as the flatter distribution of $p_{\mathrm{on}\to\mathrm{off}}$ in the central region.

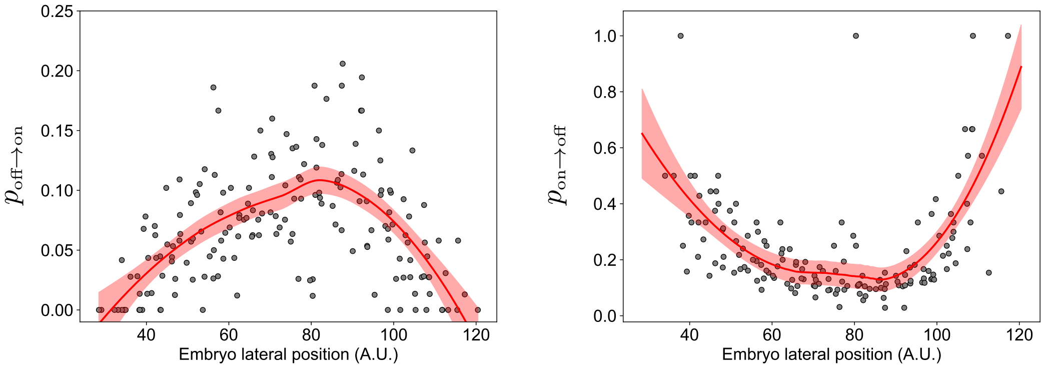

Figure S1: LOESS fit to inferred single-cell transition probability parameters for the *ush* gene against embryo laternal position.

In order to quantify the uncertainty associated with the parameter estimate for each cell, the confidence interval for a binomial proportion was calculated for each cell using the *proportion_confint* function from the Python *statsmodels* library. Figure S2 shows these error bars plotted on the same

single cell data as the previous figure. While there is very large uncertainty associated with these estimates, the general spatial trend in the estimates can still be detected.

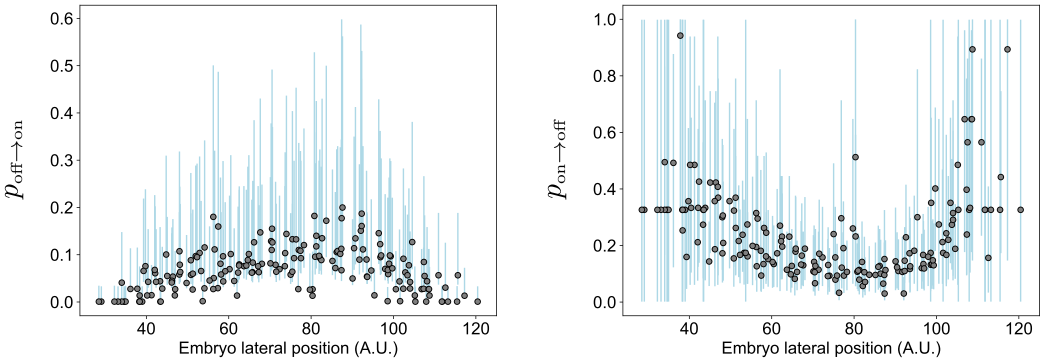

Figure S2: 95% confidence intervals for inferred single-cell transition probability parameters for the *ush* gene against embryo laternal position.
